# Engineering Neprilysin Activity and Specificity to Create a Novel Therapeutic for Alzheimer’s Disease

**DOI:** 10.1371/journal.pone.0104001

**Published:** 2014-08-04

**Authors:** Carl I. Webster, Matthew Burrell, Lise-Lotte Olsson, Susan B. Fowler, Sarah Digby, Alan Sandercock, Arjan Snijder, Jan Tebbe, Ulrich Haupts, Joanna Grudzinska, Lutz Jermutus, Christin Andersson

**Affiliations:** 1 Antibody Discovery and Protein Engineering, MedImmune, Cambridge, United Kingdom; 2 Discovery Sciences, AstraZeneca R & D, Mölndal, Sweden; 3 Global Drug Discovery, Global Biologics, Bayer HealthCare AG, Cologne, Germany; 4 CNS&P iMed, AstraZeneca R & D, Södertälje, Sweden; National Institute for Medical Research, Medical Research Council, London, United Kingdom

## Abstract

Neprilysin is a transmembrane zinc metallopeptidase that degrades a wide range of peptide substrates. It has received attention as a potential therapy for Alzheimer’s disease due to its ability to degrade the peptide amyloid beta. However, its broad range of peptide substrates has the potential to limit its therapeutic use due to degradation of additional peptides substrates that tightly regulate many physiological processes. We sought to generate a soluble version of the ectodomain of neprilysin with improved activity and specificity towards amyloid beta as a potential therapeutic for Alzheimer’s disease. Extensive amino acid substitutions were performed at positions surrounding the active site and inner surface of the enzyme and variants screened for activity on amyloid beta 1–40, 1–42 and a variety of other physiologically relevant peptides. We identified several mutations that modulated and improved both enzyme selectivity and intrinsic activity. Neprilysin variant G399V/G714K displayed an approximately 20-fold improved activity on amyloid beta 1–40 and up to a 3,200-fold reduction in activity on other peptides. Along with the altered peptide substrate specificity, the mutant enzyme produced a markedly altered series of amyloid beta cleavage products compared to the wild-type enzyme. Crystallisation of the mutant enzyme revealed that the amino acid substitutions result in alteration of the shape and size of the pocket containing the active site compared to the wild-type enzyme. The mutant enzyme offers the potential for the more efficient degradation of amyloid beta *in vivo* as a therapeutic for the treatment of Alzheimer’s disease.

## Introduction

Neprilysin, or neutral endopeptidase (NEP), is an integral type II membrane-bound zinc-dependent peptidase of approximately 750 amino acid residues [1] that degrades a number of physiological peptides that are involved in processes such as blood pressure regulation and nociception. NEP is composed of an ectodomain, which contains the catalytic site and belongs to the M13 family of peptidases/proteases, a transmembrane domain and a short intracellular domain. The structure of the ectodomain is composed of two largely α-helical domains (domains 1 and 2) that are arranged to form a central spherical water-filled core that contains the active site of the enzyme. The larger N-terminal domain (domain 1), which is structurally related to the bacterial protease thermolysin, contains a single zinc atom that is critical for peptidase activity [2]–[4] and is coordinated by His and Glu residues [5]. Whilst the substrate specificity of the enzyme is quite broad, NEP has a strong preference for peptides over larger proteins. This specificity seems to result from the enclosed catalytic chamber and size-restricted access to that chamber. Within peptide sequences cleavage is usually at the N-terminal side of a hydrophobic residue, with a strong preference for Phe or Leu at the P1’ position [6] and corresponds with a deep lipophylic cavity at subsite S1’. While structures of NEP in complex with different inhibitors display similar main-chain conformation, large ligand induced flexibility is observed for the side chains that form the substrate binding pockets (such as Arg^102^, Phe^106^, Arg^110^ and Trp^693^), which may explain the broad substrate specificity of the enzyme [5], [7], [8].

Amongst the peptides cleaved by NEP are a variety of physiologically relevant peptides such as enkephalins, tachykinins, and natriuretic peptides [9], where NEP may play a role in their degradation to limit activity: In hypertensive rats inhibition of NEP results in a lowering of mean arterial blood pressure and an increased natriuresis suggesting increased activity of natriuretic peptides [10]. Similarly, inhibition of NEP in normal rats results in a potentiation of exogenously applied atrial natriuretic peptide (ANP) activity, increasing natriuresis and lowering of blood pressure [11]. The antinociceptive activity of endogenously administered enkephalins could also be increased through blockade of NEP activity [12]. NEP has also been implicated in lipid and glucose metabolism, and suppression of NEP activity by genetic or pharmacologic means leads to late-onset obesity in mice [13], suggesting a role in regulating orexigenic peptides such as neuropeptide Y, a known NEP substrate [14].

NEP has attracted interest as a potential therapeutic to treat Alzheimer’s disease through its ability to degrade the peptide amyloid beta (Aβ) [15], [16]. In animal models manipulation of the levels of brain NEP has been shown to have a significant effect on Aβ levels. Mice deficient in the NEP gene show defects in their ability to degrade both endogenous and exogenously applied Aβ [17] and inhibition of NEP using thiorphan or phosphoramidon results in accumulation of Aβ in rats [18], rabbits [19] and non-human primates [20]. Over-expression of NEP in the brain through transgenic [21] or viral transduction [22]–[24] results in a lowering of Aβ levels in the brain. However, because of its broad substrate specificity, and known effects on blood pressure, increasing the levels of NEP through regular dosing to degrade Aβ has the theoretical risk of increasing the degradation of other physiological peptides and generating unwanted side effects. It would therefore be preferable to work with an enzyme that has higher specificity and/or activity on the desired substrate. It has previously been demonstrated that NEP is amenable to protein engineering to alter its activity and specificity [25]. Mutation of two key residues located in the substrate binding pockets adjacent to the active site resulted in NEP variants that had altered specificity towards Aβ, insulin-b chain and leu-enkephalin. However, these alterations in specificity were also accompanied by an overall reduction in the catalytic activity of the enzyme. To generate a variant of NEP that may have greater therapeutic use it would be preferable to increase its activity at the desired substrate, Aβ, and reduce activity at peptides where the potential for unwanted side effects exist.

We performed extensive mutagenesis on the solvent accessible core of the protein and screened the resulting NEP variants for their ability to degrade Aβ1–40, Aβ1–42 and a selection of other physiological peptides. We identified a panel of variant NEP molecules with increased activity in cleavage of Aβ1–40 and Aβ1–42 and reduced activity on a panel of other peptide substrates. We selected one variant for further analysis and demonstrate it has improvement in its ability to degrade Aβ1–40 and 1–42 and up to 3,000-fold reduced activity on the other peptides. Further characterisation revealed a change in the cleavage sites within Aβ1–40 and an altered sensitivity to inhibitors. We crystallised this variant and showed that long range structural changes combined with changes in the shapes of the substrate binding pockets, are likely to be responsible for the alterations in substrate specificity. The significantly enhanced Aβ cleavage and reduced activity on other peptides make this variant an attractive molecule for the potential treatment of Alzheimer’s disease.

## Materials and Methods

### Cloning, expression and purification of NEP variants

To generate NEP variants for screening for improved activity and specificity on Aβ, DNA encoding the extracellular domain (amino acids 52–749) of wild type human NEP was cloned into the yeast expression vector pYES2. The expression construct was assembled to have the mating factor alpha leader, a triple HA-tag and a Gly-Ser dipeptide linker at the N-terminus of NEP. To attempt to alter the activity and specificity of NEP 134 amino acids lining the active site were identified from the crystal structure [5] and a library of variants was generated by site-directed mutagenesis at each of these positions. In subsequent rounds of screening, mutations that resulted in improved activity on Aβ and/or improved specificity over other peptides were combined.

To screen for improved specificity towards Aβ, wild-type NEP and the variants were expressed in *Saccharomyces cerevisiae* YMR307w (EUROSCARF) cultured in SC medium (yeast nitrogen base; Becton Dickinson) or CSM-Ura medium (MPBio) supplemented with 0.5% (w/v) casein hydrolysate, 200 mM HEPES (pH 7.0) and 2% (w/v) galactose for induction of expression for 55–70 h at 30°C. CSM-Ura medium lacks uracil to enable selection of strains carrying the expression construct. Purification of HA-tagged NEP variants was performed by immunoaffinity chromatography with a monoclonal antibody specific for the HA-tag [26]. Eluted proteins were buffer-exchanged into 50 mM HEPES (pH 7.0) containing 300 mM NaCl.

Selected NEP variants were expressed in mammalian cell culture to allow their kinetic characterisation. DNA encoding amino acids 52–749 of human NEP variants was amplified by PCR. Sequences encoding an N-terminal 10-His tag and the human immunoglobulin heavy chain leader sequence were added by successive rounds of PCR and the resulting constructs were cloned into pENTR (Invitrogen) before being further sub-cloned into pDEST12.2 (Invitrogen) for expression in mammalian cells.

10-His NEP variants were expressed transiently in CHO suspension cells and secreted into the medium. Expression constructs were used to transfect cells using Polyethylenimine Max (Polysciences) and cells were cultured in CD-CHO (Invitrogen) for 10 days at 37°C, 5% CO_2_ and 80% relative humidity with shaking at 140 rpm. The supernatant was harvested, concentrated, and exchanged into 2×phosphate buffered saline (PBS) (2.2 mM KH_2_PO_4_, 310 mM NaCl, 5.9 mM Na_2_HPO_4_, pH 7.4) using cross-flow dialfiltration with a 30 kDa membrane. 10His-NEP proteins were purified first by nickel affinity chromatography using a 5 ml HisTrap excel column (GE Healthcare), followed by size exclusion chromatography using a Superdex 200 HiLoad 16/60 (GE Healthcare) equilibrated with PBS (1.1 mM KH_2_PO_4_, 155 mM NaCl, 3.0 mM Na_2_HPO_4_, pH 7.4) and eluted with an isocratic flow. Protein concentration was measured by absorbance at 280 nm using a UV Vis spectrophotometer and purity was estimated using SDS PAGE. Typically the yield was 2–20 mg/L of pure protein (>95% pure).

To produce protein for crystallisation, N-terminally His-tagged NEP G399V/G714K (NEPv) was cloned into PICZαA and expressed in *Pichia pastoris* strain GS115 *his4* His-, Mut+ (Invitrogen). A clonal line strongly expressing NEPv was selected for fermentation in BMGY medium supplemented with 12 mL/L PMT1 salts and 0.04 g/L L-histidine or basal salt minimum (BSM) medium (1.7 mM CaSO_4_, 26 mM K_2_SO_4_, 15 mM MgSO_4_, 5% (w/v) glycerol, 24 mM (NH_4_)_2_SO_4_, 12 g/L Na-hexametaphosphate, 12 ml/L PMT1 salts, 0.04 gr/L L-histidine, and antifoam). Fermentors were inoculated at OD of 1 and grown at 30°C with the addition of L-histidine at 10 g/L/day. After intitial glycerol depletion, a constant glycerol feed was applied at ∼18 mL/h/L until cell density reached approximately 200 g/L. Subsequently, a three hour starvation period was applied, during which the temperature was gradually reduced to 20°C. Protein production was induced by the addition of methanol feed at 2 mL/L/h, which was increased to 3.5 mL/L/h after 3 h. The supernatant containing secreted NEPv protein was harvested after 24 hrs (BMMY medium) or 48 hrs (BSM medium). NEPv was purified by nickel affinity chromatography using a 5 mL His Trap HP column and dialysed into 18 mM KPO_4_ (pH 6.0) containing 500 mM NaCl. The protein was deglycosylated using 833 Units EndoH (New England Biolabs) per mg NEPv with overnight stirring at 4°C, followed by size-exclusion chromatography using a Superdex200 prep grade 26/600 column. Three main species of NEPv (82002, 82204 and 82408 Da) were observed by mass spectrometry.

### Peptide cleavage assays

To screen NEP variants for improved activity on and specificity towards Aβ a high-throughput assay was developed where cleavage activity on Aβ1–40 or 1–42 and eight off-target peptides (see Table S1) was determined by fluorescence polarisation. Peptide substrates were labelled with DY-505 or DY-647 at the N-terminus and biotin at the C-terminus. The biotin serves for increasing the molecular size of uncleaved peptides after addition of strepavidin, thereby increasing the assay window and measurable signals. Peptide cleavage assays were performed by incubating NEP variant samples in a microtitre plate containing assay solution composed of 60 nM peptide substrate in 50 mM HEPES (pH 7.4) containing 150 mM NaCl and 0.05% Pluronic F-68 (Sigma). Assays were incubated at 37°C for appropriate lengths of time to allow 5 to 90% turnover of substrate, after which reactions were stopped by the addition of an equal volume of 1.2 µM streptavidin (Calbiochem). In the case of ANP and endothelin the stop solution also contained 10 mM dithiothreitol. The extent of peptide cleavage was determined by measuring the fluorescence polaristion of the stopped reactions on a plate reader (Tecan) with an appropriate set up of polarisation filters.

Since the concentrations of each enzyme variant was constant for all measurements and all substrate concentrations were << K_M_ values, the relative rate of peptide cleavage, k_app_, was proportional to k_cat_/K_M_. This allowed NEP variants to be ranked based on their relative k_cat_/K_M_ ratios on Aβ and the other peptide substrates.

For detailed kinetic characterisation of peptide cleavage by NEP, a similar assay to that above was used with modifications based on a published method [27]. Peptides were synthesised by Bachem using 9-fluorenylmethyloxycarbonyl solid-phase peptide synthesis and were labelled with a N-terminal 5(6)FAM via a ε-aminocaproic acid linker and a C-terminal biotin via a lysine linker (Table S1). Purified peptides were supplied as trifluoroacetate salts with a purity ≥95%. The assay was performed in a 96-well microtitre plate and contained 50 mM HEPES (pH 7.4), 100 mM NaCl, 0.05% (w/v) BSA (Buffer A), 1–200 µM peptide substrate and 1–500 nM NEP protein. Reactions were incubated at 37°C before being stopped at various time points between 2 and 360 min by transferring 5 µL aliquots to 245 µL Buffer A containing 2 mM 1,10-phenanethroline (Sigma) and 2 µM avidin (Invitrogen). For peptide substrates containing disulphide bonds, 1 mM tris(2-carboxyethyl)phosphine (Thermo Scientific) was added to the stop solution. The fluorescence polarisation of the resulting solution was measured on a Victor plate reader and the amount of substrate cleaved was determined with reference to substrate-only controls with and without avidin. Initial rates were obtained by linear regression of the linear regions of time courses. Enzyme velocity was plotted as a function of substrate concentration and the Michaelis-Menten equation was used to fit the data, giving the parameters k_cat_ and K_M_.

Kinetic parameters for cleavage of endothelin-1 by NEP were determined using a stopped assay in which the amount of substrate cleaved was measured using reversed-phase HPLC (RP-HPLC). Endothelin-1 was incubated at 12.5–200 µM in the presence of wild type NEP or NEPv and reactions were stopped after 10 min at 37°C by the addition of an equal volume of 1∶1∶1 water:acetonitrile:TFA containing 2 mM tris(2-carboxyethyl)phosphine (Thermo Scientific). RP-HPLC spectra were recorded using an Agilent 1260 Infinity system. Injections of 20 µL were made for each sample and analytes were separated by elution on an Agilent Polaris C8-A stationary phase column (4.6×100 mm, 3 µm) at 1.5 mL min^−1^ using a linear binary gradient of 10–90% acetonitrile in water with 0.1% (v/v) TFA over 15 minutes at 40°C. Signal intensity for intact endothelin-1 was monitored at 210 nm and relative peak area was determined by manual integration of the baseline. Kinetic parameters were derived from the activity data as described above.

The effect of inhibitors on NEP activity was determined using the above fluorescence polarisation assay to measure the rate of Aβ1–40 cleavage with a substrate concentration of 10 µM, an enzyme concentration of 20 nM and inhibitor concentrations in the range 2 nM to 100 µM. The NEP inhibitors phosphoramidon and thiorphan were purchased from Sigma and were dissolved to 10 mM stock solutions in DMSO before being diluted in Buffer A. Activity values were plotted as percentage of uninhibited control against log_10_ [inhibitor] and the log(inhibitor) vs. normalised response in GraphPad Prism was used to fit the data to generate IC_50_ values.

### Mass spectrometry

Unlabelled Aβ1–40 and bradykinin were supplied as TFA salts by Bachem. Cleavage of the peptide substrates was assessed following incubation of wild-type NEP or mutants with 10 µM Aβ1–40 or 2 µM bradykinin in 50 mM HEPES (pH 7.4) containing 150 mM NaCl at 37°C. Reactions were stopped at 0, 10, 60 and 360 min by the addition of 0.5% (v/v) trifluoroacetic acid. Stopped cleavage reactions were purified on C18 Zip-tips (Millipore), eluting directly into MALDI matrix solution (10 mg/ml α-cyanohydroxycinnamic acid dissolved in 50∶50∶0.1 water:acetonitrile:TFA). Volumes of 1 µL were applied to a MALDI target and air dried. Mass Spectra were acquired on an AB Sciex 4800 ToF/ToF mass spectrometer operating in positive reflector mode with external calibration on a peptide mixture. Peptides appearing in the digests were analysed by MS/MS with collision-induced dissociation, and identified by manual assignment in comparison with the Aβ1–40 peptide sequence.

### Crystallization and structure determination of NEP G399V/G714K

Purified NEP G399V/G714K was concentrated to 10 mg/ml in 25 mM Tris-HCl (pH 7.0), 150 mM NaCl and 2 mM MgCl_2_. Phosphoramidon was added to 2 mM from a 50 mM DMSO stock and the protein-ligand complex was incubated on ice for 30 min. Diffraction-quality crystals were obtained by hanging-drop vapor diffusion at 20°C by mixing 1 µl of protein-ligand solution with 1 µl of reservoir solution containing 100 mM HEPES (pH 7.0), 22% (w/v) PEG3350 and 200 mM NaCl. Rod shaped crystals appeared after 2 weeks. Crystal size and appearance was further improved by streak seeding. The crystals were cryo protected in reservoir solution supplemented with 20% glycerol and flash frozen in liquid nitrogen.

Crystallographic data were collected at 100 K to 2.15 Å resolution at the I04-1 beamline at the Diamond Light Source on a ADSC Q315 CCD detector and processed with MOSFLM [28]) and SCALA [29]. The space group was P3_2_21 with one NEP-phosphoramidon complex in the asymmetric unit, and 5% of the reflections were used to calculate *R*_free_. The structure was solved by molecular replacement using MOLREP [30] with coordinates from PDB ID 1dmt as a search model. The phosphoramidon ligand was well defined in the difference electron density. Crystallographic refinement was performed with a combination of Refmac5 [31] and autoBUSTER [32], Coot [33] was used for model building. X-ray data collection and refinement statistics are listed in Table 1. The crystal structure has been deposited in pdb under the accession code 4CTH. Figures showing structural representations were prepared using PyMOL [34].

**Table 1:** X-ray data collection and refinement statistics of NEPv in complex with phosphoramidon.

Data collection	
Space group	P3_2_21
Unit cell dimensions	
(Å)	a = b = 108.80, c = 112.94
(°)	α = β = 90.0, γ = 120.0
Resolution range (Å)	43.48–2.15 (2.21–2.15)
No. of observations	390649
No. of unique reflections	42405
Data redundancy	9.2 (8.0)
Data completeness (%)	99.8 (99.8)
< I/σ(I)>	16.4 (2.0)
*R*_merge_ (%)	9.8 (127)
**Refinement**	
Resolution range (Å)	31.48–2.15 (2.21–2.15)
*R*_work_ (%)	20.9 (41.0)
*R*_free_ (%)	26.1 (44.3)
Wilson *B*-factor (Å^2^)	42.4
Overall mean *B*-factor (Å^2^)	47.5
No. of atoms	
Protein atoms	5611
Heterogen atoms	453
Solvent atoms	325
r.m.s.d. values	
Bond lengths (Å)	0.010
Bond angles (°)	1.09
Ramachandran statistics (%) (PROCHECK (Laskowski, 1993))	
Most favoured + additional allowed	99.8
Disallowed	0.2

## Results

### Engineering NEP for increased activity and specificity at Aβ

**Figure 1:**
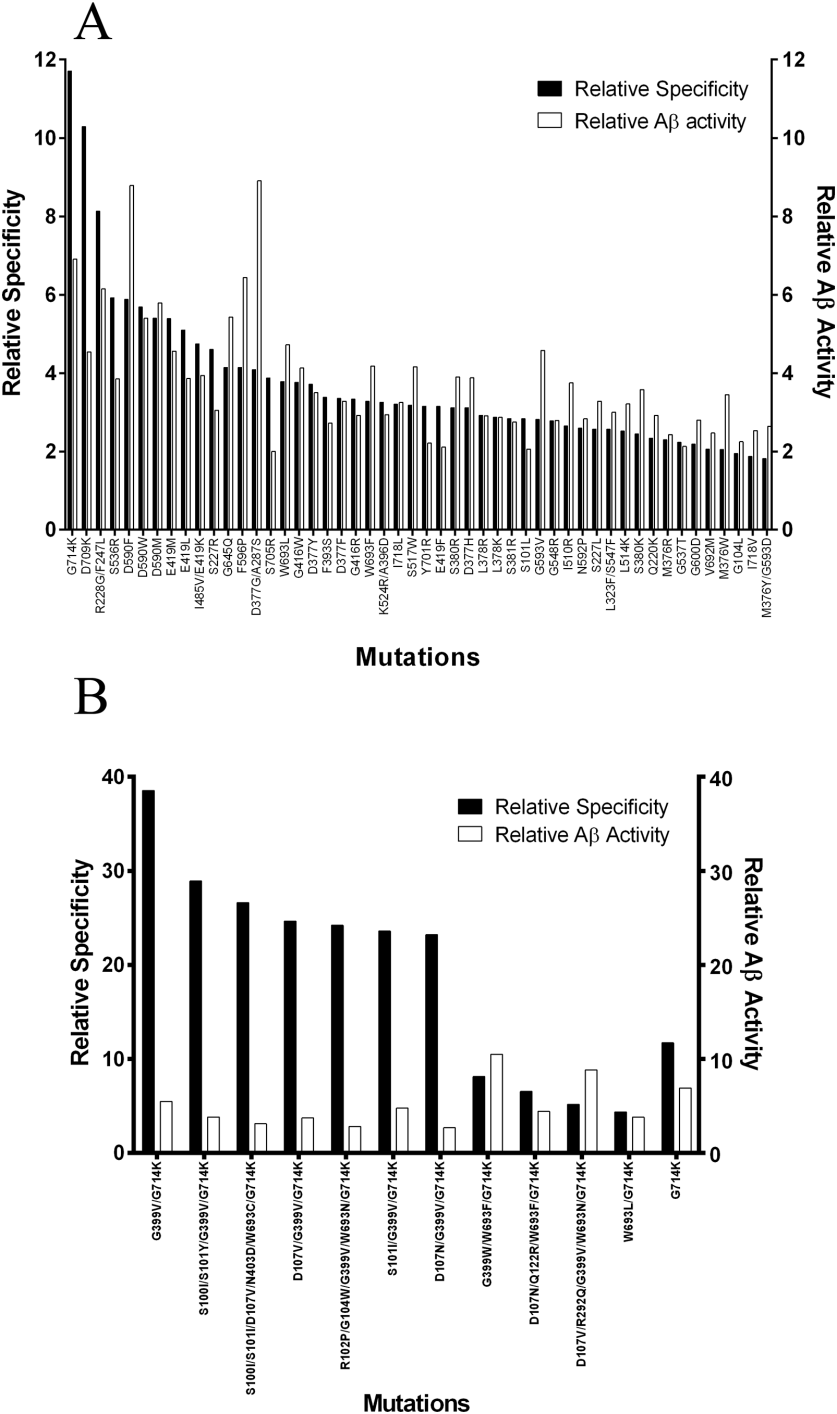
Relative activities of the mutants identified after screening. For each variant the relative activity at Aβ divided by the relative activity on the off target peptides is shown in the black bar and the relative activity at Aβ is shown in the open bar. Mutants are ranked by relative activity at Aβ divided by the relative activity on the off target peptides after the first **(A)** and second **(B)** rounds of screening. Each bar represents and average of four readings for each variant on Aβ alone, or Aβ and up to 9 other peptides. Error bars are not shown due to the complex nature of the propagation of errors calculation for the relative specificity change where activity on up to 11 different peptides was measured for both wild-type and variant enzymes. For the first round of screening only the top 50 mutants are shown. Despite the intention of introducing only single amino acid substitutions into the enzyme in the first round a number of the variants with improved specificity had acquired two mutations. Activities are relative to the wild-type enzyme.

Screening of the variants generated from the first round library resulted in the identification of NEP mutants with altered activity on all of the peptide substrates. To rank these variants the change in activity on the Aβ1–40 peptide was divided by the average change in specificity on the other peptides and then by the specificity of the wild-type enzyme. The mutants were then ranked according to these scores and compared to the change in activity on Aβ1–40 (Figure 1A). Improvements in activity on Aβ of up to approximately 9-fold and specificity changes of up to approximately 12-fold were observed. The DNA encoding each of the variants with altered specificity was sequenced and the mutations resulting in these changes identified. A wide range of mutations at numerous positions within the central core of the enzyme affected the activity on Aβ and the other peptides. In particular, mutations at positions 590 and 377 where replacement of the aspartate at these positions with one of a number of amino acids with larger, hydrophobic side chains resulted in improvements in activity and specificity. At many other positions only a single amino acid substitution was detected as having improved specificity, and some of these provided the largest gains observed. The greatest change in specificity was observed with a change at position 714 from glycine to lysine, a change that introduces both a positive charge and a larger side chain. In the high throughput screening this change improved specificity by ∼12-fold and activity on Aβ by ∼7-fold. There were several other positions where introduction of a lysine or arginine resulted in improvements in activity and specificity, notably positions 705 and 709 suggesting the introduction of a positive charge in this area was beneficial for the binding or cleavage of Aβ and inhibitory for the binding and/or cleavage of the other peptides. From this analysis mutations that improved activity on Aβ1–40 and reduced activity on the other peptides were identified and used to design a second library. Because of its strong specificity change G714K was fixed and randomly combined with other beneficial mutations to generate the second round library of variants. Following screening on the peptide panel, the variants were again ranked and improvements in specificity of around 40-fold over wild-type were now observed. However, the variants did not demonstrate improvements in the cleavage of Aβ over those seen on round 1 and a smaller number of hits were detected than at round 1, (Figure 1B). While introduction of further mutations resulted in additional improvements in specificity, this was accompanied by a marked decrease in the stability of the enzyme with the majority of the expressed material being aggregated (results not shown).

### Increased specificity of NEP mutants towards Aβ peptide

**Figure 2:**
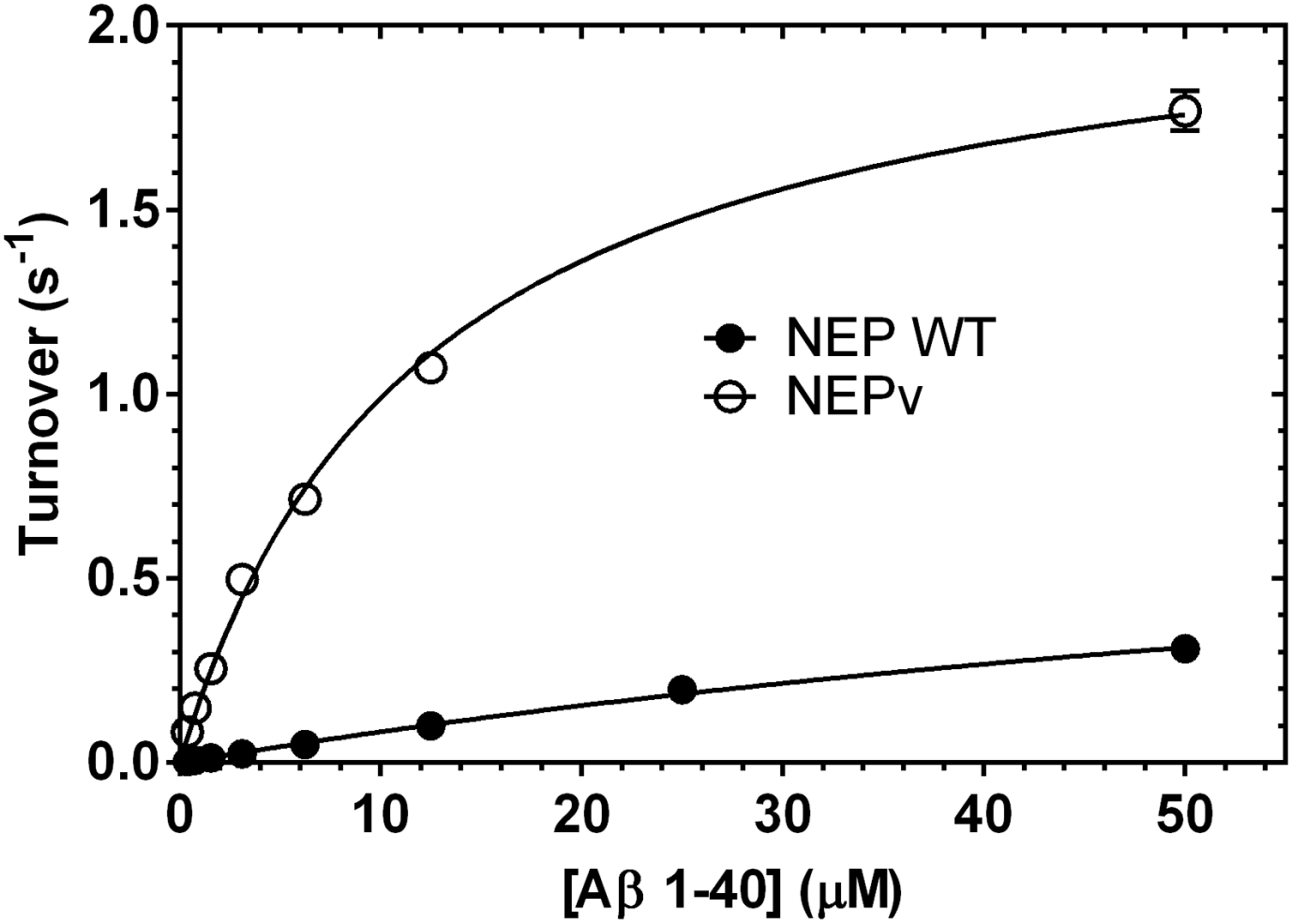
Michaelis-Menten profiles for cleavage of Aβ1–40 by wild-type NEP and NEPv. Aβ1**–**40 cleavage activity was determined at 0.4**–**50** µ**M substrate with 40****nM wild-type NEP (•) or 10****nM NEPv (○). Activity data were normalised to enzyme concentration and plotted against substrate concentration, and the Michaelis-Menten equation was used to fit them. Error bars represent the spread of two replicate data points. NEPv showed **∼**20-fold increased catalytic efficiency on Aβ1**–**40 compared to wild-type NEP. The limited solubility of FAM- and biotin-labelled Aβ1**–**40 in aqueous buffers precluded the use of substrate concentrations above 50** µ**M in the assay.

**Figure 3:**
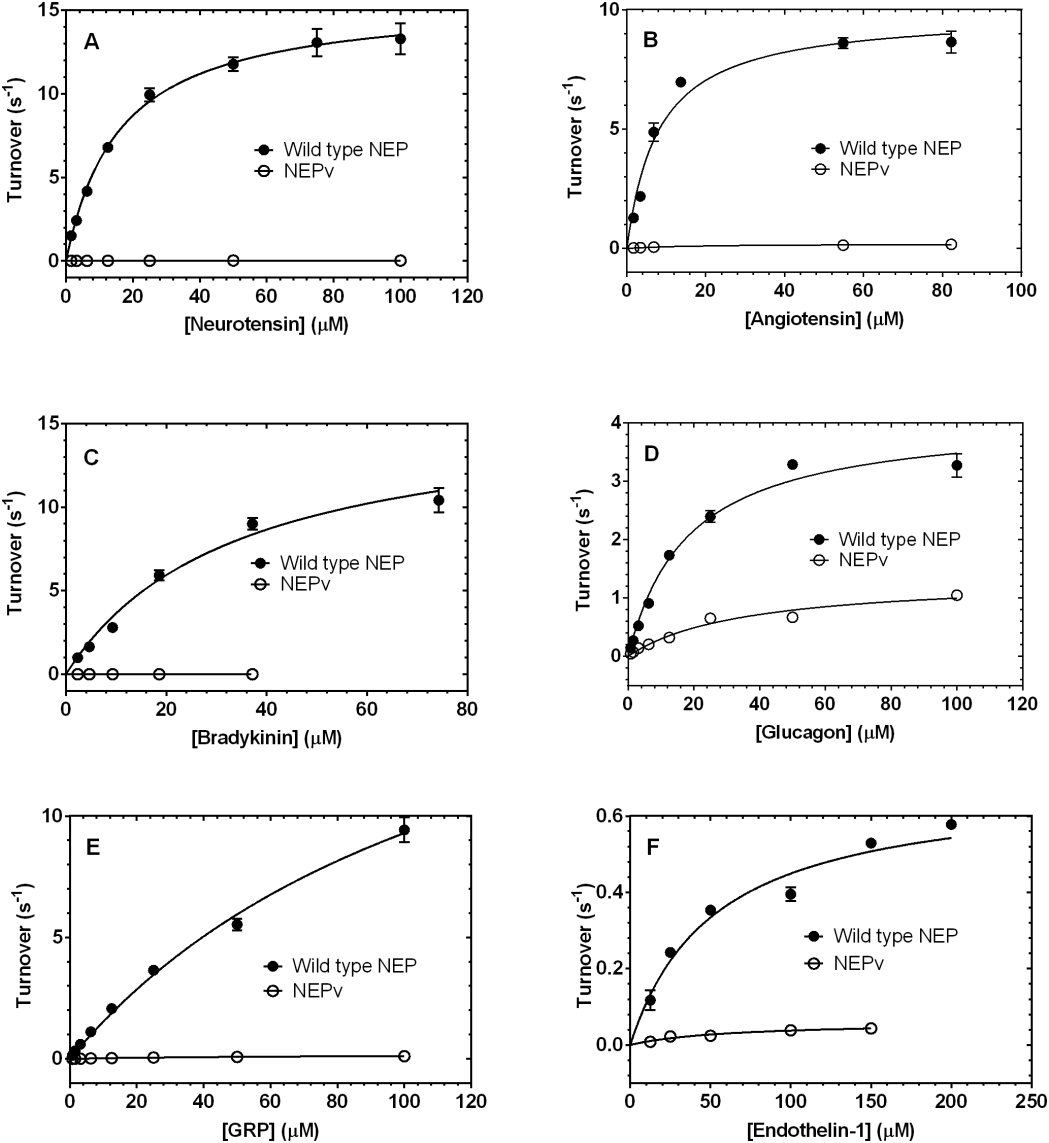
Cleavage activity of wild-type NEP and NEPv on selected off-target peptides. Cleavage activity wild-type NEP (•) and NEPv (○) was determined on neurotensin (A), angiotensin (B), bradykinin (C),glucagon (D), GRP (E) and endothelin-1 (F) at a range of substrate concentrations. Activity data were normalised to enzyme concentration and plotted against substrate concentration, and the Michaelis-Menten equation was used to fit them. Error bars represent the spread of two replicate data points. The data are representative of at least two replicate experiments.

The lead variant at round 2, NEP G399V/G714K (NEPv), was selected for further characterisation as it had increased activity on Aβ1–40 and Aβ1–42 and reduced activity on a panel of other natural substrates of the enzyme. In order to determine the magnitude of the increase in specificity towards Aβ, kinetic parameters for cleavage of Aβ1–40 and 16 off-target peptides were measured and compared to those of the wild-type enzyme (Figure 2 and Figure 3). On Aβ1–40 wild-type NEP had a k_cat_ of 0.83±0.1 s^−1^ and a K_M_ of 104±20 µM (Table 2). These values gave a k_cat_/K_M_ of 8.0×10^3^ M^−1^s^−1^. The value of k_cat_ measured for human wild-type NEP was consistent with previous reports for the rabbit enzyme, although the K_M_ value was ∼8-fold higher (Leisring *et al.* 2003). However, the difference in K_M_ values may be due to inherent differences between the human and rabbit enzyme or may result from difference in the preparation of the proteins. The k_cat_ of NEPv was increased ∼2-fold relative to that of wild-type NEP to 2.1±0.1 s^−1^ while K_M_ was reduced to 14±1 µM. These changes led to an increase in k_cat_/K_M_ to 1.5×10^5^ M^−1^s^1^, an increase of 18.5-fold over wild-type The cleavage of Aβ1–40 by wild-type NEP and NEPv followed normal Michaelis-Menten kinetics at substrate concentrations between 0.4 and 50 µM (Figure 2). Due to the limited solubility of FAM-Aβ1–40-biotin it was not possible to use substrate concentrations above 50 µM in this assay. Additionally, it was not possible to generate accurate kinetic data for the degradation of Aβ1–42 due to its propensity to aggregate, however, we were able to demonstrate a very similar improvement in NEPv’s activity on Aβ1–40 and Aβ1–42 using an endpoint assay (Figure S1).

**Table 2:** Steady-state kinetic parameters for cleavage of Aβ1**–**40 and off-target peptides by wild-type NEP and NEPv.

Variant	Substrate	*k_cat_* (s^−1^)*	*K_M_* (µM)*	*k_cat_/K_M_* (M^−1^s^−1^)	Fold change in *k_cat_/K_M_* #
**wild-type NEP**	Aβ 1–40	0.83±0.1	104±20	8.0×10^3^	-
	Neurotensin	13±0.6	16±2	8.2×10^5^	-
	ANP	7.1±0.2	8.4±0.8	9.1×10^5^	-
	GLP-1	3.3±0.3	24±4	1.4×10^5^	-
	Angiotensin	8.2±0.3	8.6±0.9	9.7×10^5^	-
	Bradykinin	16±1	32±5	4.8×10^5^	-
	Glucagon	4.5±0.4	25±5	1.8×10^5^	-
	Somatostatin 28	9.1±0.4	31±3	1.6×10^5^	-
	Somatostatin 14	32±1	7.4±0.9	4.4×10^6^	-
	Neurokinin A	17±0.7	9.3±1	1.7×10^6^	-
	Neurokinin B	20±0.8	22±2	9.2×10^5^	-
	Nociceptin	11±0.3	20±1	5.7×10^5^	-
	α-endorphin	8.9±0.08	24±0.5	3.9×10^5^	-
	γ-endorphin	4.8±0.2	13±1	3.8×10^5^	-
	Substance P	18±0.6	16±1	1.1×10^6^	-
	Arg-vasopressin	2.8±0.2	6.2±1	4.5×10^5^	-
	GRP	26±4	150±30	1.7×10^5^	-
	Endothelin-1	0.68±0.06	53±10	1.3×10^4^	-
**NEPv**	Aβ 1–40	2.1±0.1	14±1	1.5×10^5^	0.055
	Neurotensin	0.039±0.002	16±3	2.6×10^3^	320
	ANP	0.56±0.05	33±6	1.7×10^4^	53
	GLP-1	0.89±0.07	23±4	4.0×10^4^	3.6
	Angiotensin	0.14±0.007	13±2	1.1×10^4^	87
	Bradykinin	0.0055±0.001	37±10	1.5×10^2^	3200
	Glucagon	1.2±0.1	30±6	4.6×10^4^	4.2
	Somatostatin 28	0.086±0.02	47±20	1.8×10^3^	87
	Somatostatin 14	9.1±0.4	32±3	3.0×10^5^	15
	Neurokinin A	1.1±0.1	34±7	3.4×10^4^	52
	Neurokinin B	0.42±0.03	22±3	1.9×10^4^	48
	Nociceptin	14±0.9	63±7	2.2×10^5^	2.6
	α-endorphin	0.089±0.003	6.5±1	1.5×10^4^	26
	γ-endorphin	0.16±0.006	4.4±0.6	3.8×10^4^	10
	Substance P	12±0.9	92±10	1.3×10^5^	8.7
	Arg-vasopressin	0.017±0.001	22±3	9.0×10^2^	510
	GRP	0.39±0.05	120±20	3.3×10^3^	52
	Endothelin-1	0.059±0.008	57±20	1.0×10^3^	13

*the best fit values of *k_cat_* and *K_M_* were determined by non-linear regression of activity data using the Michaelis-Menten equation. Values are quoted ± the average standard error generated from non-linear regression of data from two independent experiments.

# the fold-change in *k_cat_/K_M_* is the ratio of the wild-type NEP *k_cat_/K_M_* to that of NEPv.

Kinetic parameters for cleavage of a panel of peptides by wild-type NEP and NEPv were determined (Figure 3 and Table 2). The kinetic parameters determined for wild-type NEP on these substrates agreed reasonably well with those previously reported [4], [35]. NEPv displayed significantly reduced catalytic efficiency relative to that of wild-type NEP on all of the other peptides tested. The greatest effect was observed with bradykinin, on which NEPv had 3,200-fold reduction in k_cat_/K_M_ relative to that of wild-type NEP. The reduction in catalytic efficiency was largely due to a decrease in k_cat_; the K_M_ for bradykinin was relatively unaffected suggesting that the mutations introduced into NEPv have not affected binding of the substrate to the active site. This trend was observed across the panel of off-target peptides; the majority of the reductions in k_cat_/K_M_ could be attributed to decreases in k_cat_. For example, NEPv showed reductions in k_cat_ of 60-, 67- and 340-fold on angiotensin, GRP and neurotensin, respectively, while the K_M_ values on these substrates were relatively unaffected. The reductions in catalytic efficiency were mediated by increases in K_M_ on some substrates, such as nociceptin and substance P, although the overall changes in k_cat_/K_M_ were relatively modest with these peptides. The average specificity switch (defined as the ratio of the fold change in k_cat_/K_M_ on Aβ1–40 to that on other peptides) on the panel of 16 peptides was 5,300-fold.

To investigate which of the two mutations in NEPv made a greater contribution to the increase in activity on and specificity towards Aβ, G399V and G714K single mutants were generated and characterised. Kinetic parameters for cleavage of Aβ1–40, angiotensin, bradykinin, glucagon and neurotensin by these variants were determined (Table 3). Both NEP G399V and NEP G714K showed increases in catalytic efficiency on Aβ1–40; k_cat_/K_M_ was increased by ∼6-fold for both variants, relative to that of wild-type NEP, suggesting that the two substitutions make roughly equal contributions to the increase in activity on seen with NEPv.

**Table 3:** Steady-state kinetic parameters for cleavage of Aβ1**–**40 and off-target peptides by NEP G399V and NEP G714K.

Variant	Substrate	*k_cat_* (s^−1^)*	*K_M_* (µM)*	*k_cat_/K_M_* (M^−1^s^−1^)	Fold change in *k_cat_/K_M_* #
**NEP G399V**	Aβ1–40	2.7±0.2	55±7	4.9×10^4^	0.16
	Angiotensin	0.73±0.03	3.6±0.4	2.1×10^5^	4.6
	Bradykinin	0.15±0.01	79±10	1.9×10^3^	250
	Glucagon	2.9±0.1	20±2	1.5×10^5^	1.2
	Neurotensin	1.1±0.04	12±1	1.0×10^5^	8.2
**NEP G714K**	Aβ1–40	1.9±0.2	42±0.7	4.5×10^4^	0.18
	Angiotensin	12±0.3	18±1	6.7×10^5^	1.5
	Bradykinin	7.4±0.3	120±8	6.4×10^4^	7.5
	Glucagon	5.2±0.3	42±5	1.2×10^5^	1.5
	Neurotensin	8.6±0.2	10±0.7	8.4×10^5^	0.98

*the best fit values of *k_cat_* and *K_M_* were determined by non-linear regression of activity data using the Michaelis-Menten equation. Values are quoted ± the average standard error generated from non-linear regression of data from two independent experiments.

# the fold-change in *k_cat_/K_M_* is the ratio of the wild-type NEP *k_cat_/K_M_* to that of the NEP mutant.

The G399V mutation significantly reduced catalytic efficiency on angiotensin, bradykinin and neurotensin. For example, on bradykinin NEP G399V showed a 250-fold reduction in k_cat_/K_M_ relative to wild-type NEP. The G714K mutation had much less effect on the activity towards other peptides; k_cat_/K_M_ on bradykinin was reduced by only 7.5-fold while catalytic efficiency on angiotensin, glucagon and neurotensin was relatively unaffected compared to that of wild-type NEP. These data suggest that G399 has a greater role than G714 in regulating the substrate specificity of NEP.

We next investigated whether the mutations introduced to NEP had affected its sensitivity to known inhibitors of the enzyme. To determine the potencies of inhibition of wild-type NEP and NEPv by phosphoramidon, Aβ1–40 cleavage activity was measured over a range of inhibitor concentrations (Figure S2). Phosphoramidon inhibited wild-type NEP with an IC_50_ of <10 nM (Table S2). Because of the relatively low activity of wild-type NEP on Aβ1–40 it was not possible to use an enzyme concentration lower than 20 nM in the assays and therefore the lowest IC_50_ value that could be measured was 10 nM. Phosphoramidon inhibited NEPv with an IC_50_ of 21 µM, a potency >2000-fold lower than for wild-type NEP. Although the observed reduction in potency may be partially explained by the increased catalytic efficiency of NEPv on Aβ1–40, it nevertheless suggests that the mutant has increased active site binding selectivity. To investigate the relative roles of the G399V and G714K substitutions in this decrease in sensitivity to the inhibitor, inhibition of the NEP single mutants by phosphoramidon was characterised. As was observed with peptide cleavage activity, the G399V mutation had a greater effect than G714K on sensitivity to the inhibitor; NEP G399V was inhibited with an IC_50_ of 610 nM compared to 100 nM for G714K. These observations are consistent with the hypothesis that G399 has a greater role than G714 in controlling the selectivity of active site binding.

Similar effects were observed with thiorphan, a highly selective and potent inhibitor of NEP [36]. Wild-type NEP was inhibited with an IC_50_ of <10 nM, whereas the IC_50_ for inhibition of NEPv was 77 µM. As was observed with phosphoramidon, the G399V mutation had a greater effect than G714K on sensitivity towards thiorphan; NEP G399V was inhibited with an IC_50_ of 4.4 µM whereas the IC_50_ for NEP G714K was 75 nM (Table S2).

### Altered cleavage site preferences of NEP mutants

Although NEP is relativity promiscuous in its cleavage site sequence specificity, it does exhibit a preference for hydrophobic residues, such as Phe or Leu, in its P1’ site (MEROPS database, [37]). Wild-type NEP is able to cleave Aβ1–40 at several sites although it appears to initially degrade the substrate preferentially at Lys^16^-Leu^17^ and Phe^19^-Phe^20^ [25], [38]. Given the increased activity on Aβ1–40 and reduced cleavage of other peptides, as well as the altered binding of inhibitors to the active site observed with NEPv, it seemed possible that Aβ may be cleaved at different sites in the variant compared to wild-type NEP. To investigate this possibility, Aβ1–40 was incubated with wild-type NEP or NEPv and cleavage products were analysed after 10, 60 and 360 min by MALDI MS.

**Figure 4:**
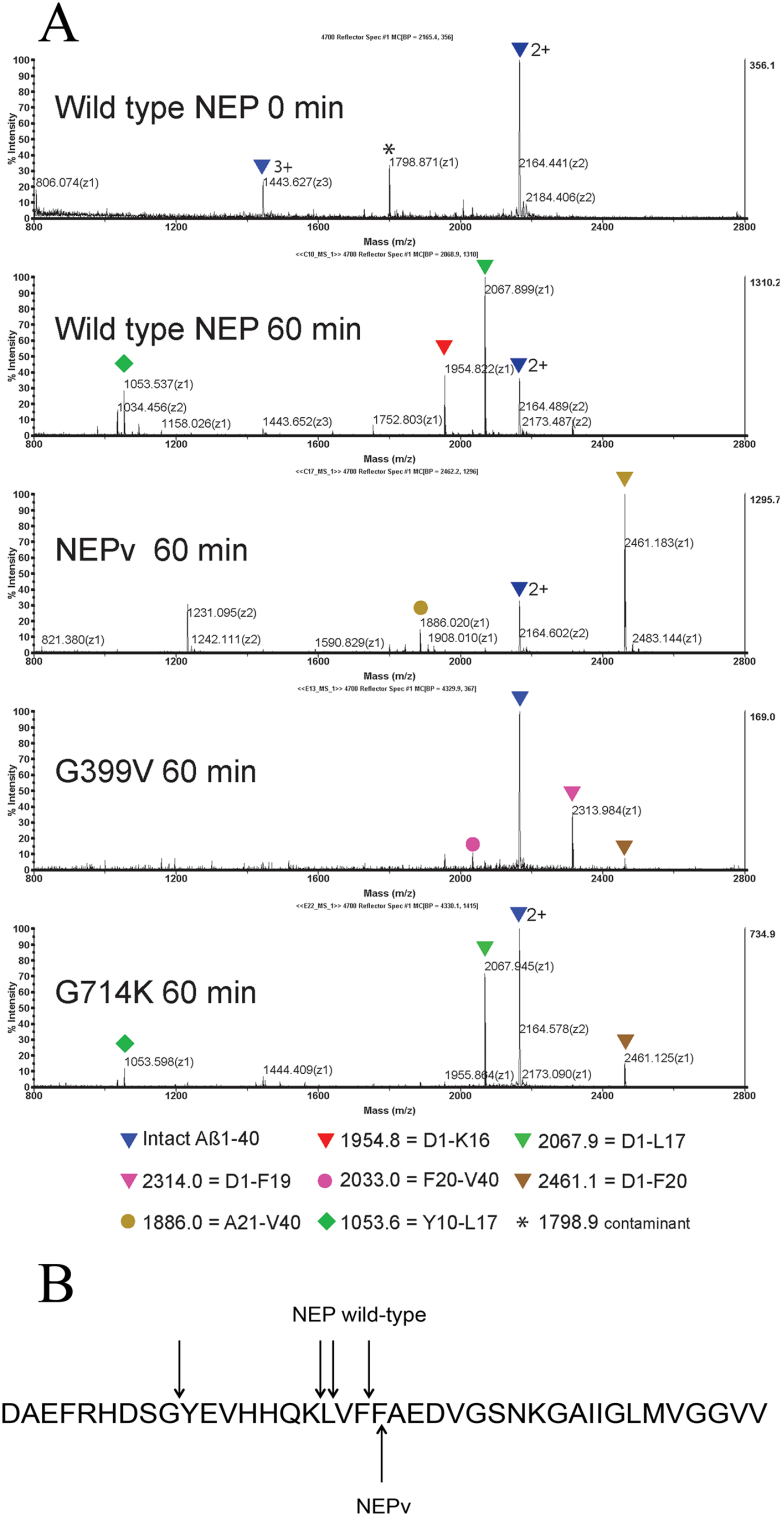
MS analysis of Aβ1–40 peptide cleaved by wild type NEP and variants. Aβ1**–**40 peptide was incubated with different sequence variants of neprilysin and the appearance of cleaved fragments was detected by MALDI mass spectrometry. A) expanded region between 800–2800**** *m/z*. Peptide identities were confirmed by MS/MS analysis and are indicated by triangles (N-terminal fragments) or circles (C-terminal fragments). B) Cleavage preference on Aβ1**–**40 exhibited by wild type NEP and NEPv. Arrows indicate the predominant sites of cleavage observed.

Wild-type NEP cleaved Aβ1–40 predominantly at Lys^16^-Leu^17^, Leu^17^-Val^18^ and Phe^19^-Phe^20^; the main cleavage fragments observed after 60 min were Asp^1^-Lys^16^, Asp^1^-Leu^17^ and Asp^1^-Phe^19^ (Figure 4). After 360 min these fragments were degraded further, accompanied by the appearance of Val^12^-Leu^17^ and Tyr^10^-Leu^17^ fragments. NEPv appeared to be more selective in its cleavage site preference; Aβ1–40 was cleaved preferentially at Phe^20^-Ala^21^, as shown by the appearance of the Asp^1^-Phe^20^ fragment after 10 min. A small amount of Asp^1^-Leu^17^ was detected after 360 min although the Asp^1^-Lys^16^ and Asp^1^-Phe^19^ fragments were not observed. Cleavage of Aβ1–40 at Phe^20^-Ala^21^ by NEP has not been previously observed [25], [27], [38] and occupancy of the P1’ site with Ala is relatively uncommon (MEROPS database, [37]). These results indicate that the mutations introduced into NEPv have fundamentally altered the cleavage site preference of the enzyme. It is interesting to note that the increase in catalytic efficiency on Aβ1–40 observed for NEPv was largely driven by a decrease in K_M_, which may result from the substrate being able to adopt an altered binding conformation at the active site.

To determine whether the change in cleavage site preference observed for NEPv could be attributed to either the G399V or G714K mutations, cleavage fragments were also analysed for the NEP single mutants. NEP G399V cleaved Aβ1–40 preferentially at the Phe^19^-Phe^20^ site, as shown by the appearance of the Asp^1^-Phe^19^ fragment after 10 min. Asp^1^-Lys^16^ and Asp^1^-Leu^17^ were also present after further incubation. NEP G399V also cleaved at Phe^20^-Ala^21^, in common with NEPv, although there was a much lower preference towards this cleavage site with the single mutant. NEP G714K cleaved Aβ1–40 preferentially at Leu^17^-Val^18^. This mutant also showed some cleavage at Phe^20^-Ala^21^, although this did not represent the major cleavage site. These results suggest that while the G399V and G714K single substitutions do alter the cleavage specificity of NEP, both substitutions are necessary for the full change in cleavage site preference observed for NEPv.

The products of bradykinin digestion by wild-type NEP and NEPv were also determined by MALDI-TOF MS. Bradykinin was cleaved at Pro^7^-Phe^8^ by both wild-type NEP and NEPv, as indicated by the appearance of the Arg^1^-Pro^7^ peptide fragment. This cleavage site was consistent with that previously reported for porcine NEP [4].

### Crystal structure of the NEP variant

**Figure 5:**
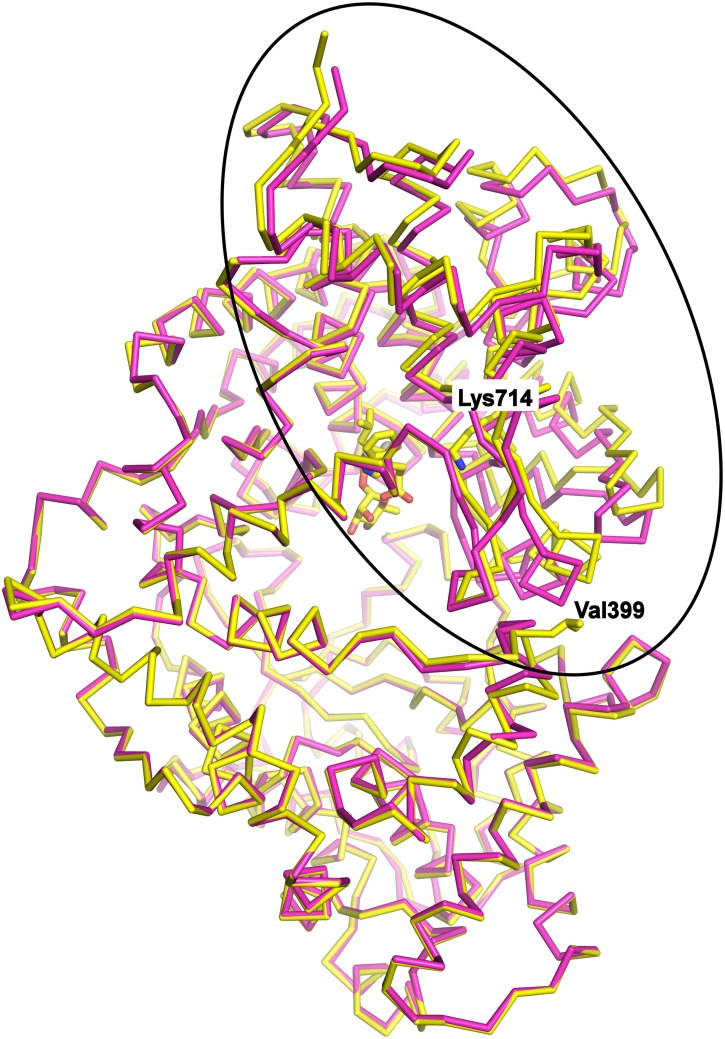
Conformational change of NEPv. Superposition of NEPv (yellow) with the published wild-type NEP structure 1dmt (magenta) (reference). The main effects of the G399V/G714K mutations are observed within the encircled part of the protein. The upper half is domain 1, the lower half is domain 2, and linker segments connecting the domains are to the left. The positions of Val399 and Lys714 are indicated and the mutants and phosphoramidon of NEPv are shown in sticks. The zinc ion is shown as a grey sphere. All pictures and structure alignments were made using Pymol.

To investigate how the two mutations had altered the structure of the enzyme the crystal structure of the extracellular domain of NEP G399V/G714K in complex with phosphoramidon was refined to 2.15 Å resolution. The double mutant crystallized in the same space group and with similar cell dimensions as the wild-type structure with phosphoramidon as described by Oefner et al., 2000 [5]. The overall structure of the double mutant NEP is similar to wild-type NEP, with two domains which are connected by three interdomain linker segments, together enclosing a large water filled cavity. Both mutated residues (G399V/G714K) have defined electron density as shown in Figure S3. Residue Lys^714^ is located in the larger domain (domain 1), close to the catalytic site. The second mutated residue Val^399^ is at a distance of 20 Å from Lys^714^, located in the smaller domain (domain 2) at the surface of the protein at the interface between the two domains. Despite the overall similarity a superposition shows substantial changes in the relative orientation of secondary structure elements predominantly in domain 1, which are illustrated in figure 5. The interdomain distance, as measured by Cα-Cα distance from residue Val^399^ in domain 2 to e. g. residue Arg^698^ in domain 1, increases by more than 2 Å (from 5.9 Å to 8.4 Å in wild-type NEP and NEPv, respectively). In domain 2, substantial main chain differences are only observed in the helix preceding the mutated Val^399^ and the loop (residues 400–404) following this residue.

**Figure 6:**
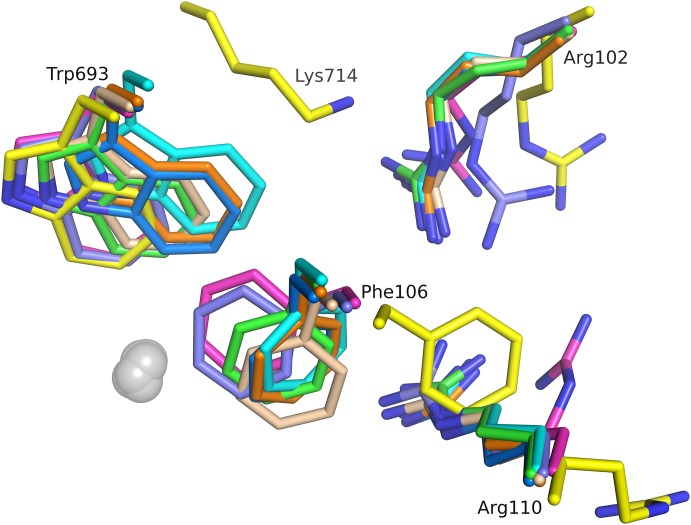
Conformational changes of residues involved in substrate binding. Superpositions of NEPv (yellow) with published structures 1 dmt (magenta), 1r1h (cyan), 2yb9 (violet), 1r1i (green), 1r1j (wheat), 1y8j (blue) and 2qpj (orange). Large ligand induced flexibility in active site. Flexible side chains include Arg102, Phe106, Arg110 and Trp693. Ligands left out for clarity. Zinc ions shown as grey spheres.

While the combined effects of the two mutations on structural changes cannot be fully deconvoluted, it is apparent that Lys^714^ protrudes in to the S2’ pocket of the enzyme and forces Arg102 away from the positions it has in wild-type NEP (Figure 6). Furthermore Lys^714^ limits the conformational flexibility of Trp^693^ and thereby reduces the plasticity and size of the S1’ pocket. Further changes in the vicinity of Lys^714^ involve Arg^110^ and Phe^106^.

**Figure 7:**
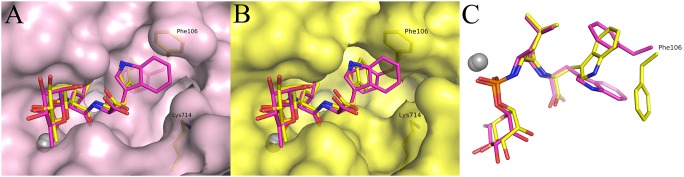
Phosphoramidon binding in wild-type and NEPv. Superposition of NEPv (yellow) and wild-type NEP (1 dmt, magenta) with phosphoramidon bound. The overall binding modes of phosphoramidon are the same, but the C-terminal extension of the molecule binds differently. A) Surface of 1 dmt and B) surface of NEPv. C) Side chain swap of Phe106 and the phosphoramidon tryptophane.

The overall binding mode of phosphoramidon in the NEP double mutant is similar to that observed in wild-type (Figure 7). The rhamnose moiety, the tetrahedral N-phosphoryl interaction with the catalytic zinc and the L-leucyl moiety in the S1’ pocket are nearly identical in both NEP forms. The C-terminal extension of the phosphoramidon binds differently. While the phosphoramidon tryptophan pyrrole retains the interaction with Val^541^ O and the stacking interaction with Phe^106^, the positions of the phosphoramidon tryptophan and Phe^106^ side chain moieties are interchanged. This different binding mode may originate from subtle changes of the main chain conformation around Phe^106^, and may favor the different rotameric orientation of Phe^106^ side chain observed in the mutant.

## Discussion

Proteases represent an attractive class of therapeutics because of their potential to irreversibly inactivate protein or peptide targets using lower amounts of the drug than would be required for stociometric binding and inactivation by molecules such as antibodies. However, because of the large substrate repertoires of many proteases, one of the key challenges to exploiting them therapeutically is the lack of specificity towards the target molecule, which may result in undesirable off-target activities. To address this challenge, protein engineering has been used to generate protease variants with increased activity on the target substrate relative to that on off-target substrates. For example, the substrate specificity of rat neurolysin has been altered by a single amino acid substitution, giving a 10-fold specificity switch [39].

NEP has been suggested as a potential therapeutic because it cleaves a wide range of physiological peptide substrates including Aβ, which is thought to be a key pathological component of Alzheimer’s disease. However, because NEP is promiscuous in its substrate preference and has relatively modest cleavage activity on Aβ, improvement in the specificity of the enzyme is required to maximize its therapeutic potential. By introducing mutations at Phe^563^ and Ser^546^, Sexton *et al.* [25] were able to alter the cleavage preference of NEP towards Aβ, insulin-b chain and leu-enkephalin, although the specificity changes observed were relatively small and accompanied by reductions in overall activity. By using a directed evolution approach, we have generated a variant of NEP that shows 20-fold increased catalytic efficiency on Aβ1–40 and up to 3.2×10^3^-fold reduced cleavage of a range of off-target peptides.

Significant increases in activity on Aβ could be achieved from single amino acid substitutions at a number of residues. For example, the G399V and G714K variants showed ∼6-fold increase in k_cat_/K_M_ on Aβ1–40. NEPv, which contains both of these mutations, had an even greater improvement in activity, with a 20-fold increase in k_cat_/K_M_ on Aβ1–40 relative to that of wild-type NEP. As well as improved activity on Aβ, NEPv displayed significantly reduced activity on a range of other peptide substrates. For example, on bradykinin k_cat_/K_M_ was reduced by 3.2×10^3^-fold giving a specificity switch of 6.4×10^4^ in favor of cleavage of Aβ. While this specificity change is impressive, switches of greater magnitude have been reported for proteases in the literature; the *E. coli* endopeptidase OmpT has been engineered to preferentially cleave Ala-Arg rather than Arg-Arg by introduction of a single amino acid substitution, giving a specificity switch in excess of 10^6^ [40]. However, it is likely that further engineering of NEP could lead to even greater improvements in specificity towards Aβ. Indeed, we were able to isolate NEP variants with greater activity than NEPv on the target substrate, although these variants had significantly lower stability and were therefore less attractive for biopharmaceutical development. Nevertheless, the enhancements in specificity towards Aβ are expected to improve the efficacy and safety profile of NEPv as a potential therapeutic for Alzheimer’s disease.

The changes in kinetic properties observed for NEPv raised the question of which of the two mutations made the greatest contribution to the increase in activity and specificity towards Aβ. Analysis of Aβ1–40 cleavage by the NEP G399V and G714K single mutants revealed that the mutations caused similar increases in activity on this substrate. However, the G399V substitution caused a much greater reduction in activity on the other peptide substrates tested, although both mutations appear to be required to achieve the full specificity switch observed for NEPv. Consistent with this observation, NEP G399V also showed a much greater reduction in sensitivity to competitive inhibitors than NEP G714K suggesting that despite its distance from the active site, Gly^399^ plays a greater role in regulating substrate binding.

As well as displaying altered substrate preference and sensitivity to inhibitors, NEPv also showed significant changes in its cleavage site preference on Aβ. NEP generally exhibits a preference for cleavage of peptide substrates on the amino terminal side of hydrophobic residues such as Phe or Leu [6] and Aβ is cleaved predominantly at Lys^16^-Leu^17^, Leu^17^-Val^18^ and Phe^19^-Phe^20^ by wild-type NEP [25], [38]. These cleavage sites were also observed for wild-type NEP in the present study. In contrast, NEPv preferentially cleaved Aβ at Phe^20^-Ala^21^ and showed little cleavage at the sites favoured by the WT enzyme. Degradation of Aβ by NEPv does not appear to be processive; the Asp^1^-Phe^20^ fragment generated by cleavage at Phe^20^-Ala^21^ was not cleaved significantly further over the course of 360 min. In contrast wild-type NEP is processive in its action, first cleaving Aβ at Leu^17^-Val^18^ and degrading the resulting fragment further to generate Tyr^−10^-Leu^17^. The lack of processivity displayed by NEPv suggests that the specificity has changed to such an extent that initial cleavage products may be released from the active site before any further degradation occurs. Thus release occurs at a greater rate, thereby accelerating k_1_, which would be consistent with the reduced K_M_ observed for the variant. Analysis of the cleavage events reported in the literature reveals that while NEP is able to cleave on the amino terminal side of Ala, this is relatively uncommon. For example, cleavage has been observed at Trp^16^-Ala^17^ of proadrenomedulin although this appears to be secondary to cleavage at Glu^8^-Phe^9^ [41].

**Figure 8:**
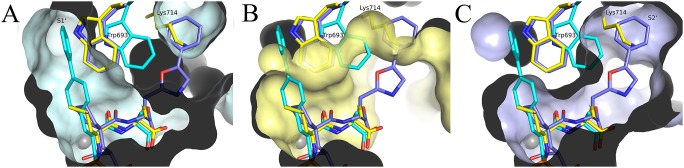
Plasticity in S1’ and S2’ substrate binding pockets. Superposition of 1r1h (cyan), NEPv (yellow) and 2yb9 (violet), illustrating flexibility of size and shape of S1’ and S2’ subsites. Protein surfaces of each complex are shown: A) 1r1h B) NEPv C) 2yb9. The inner parts of the S1’ and S2’ sub pockets will probably not be accessible in NEPv.

The structural background to the substrate promiscuity in wild-type NEP is partially illustrated by crystal structures of various inhibitors bound to NEP. Large ligand induced flexibility of amino acid side chains is observed in the substrate binding site, with particularly Trp^693^ functioning as a switch between the S1’ and S2’ pockets in wild-type NEP (Figure 8); (P1’ and P2’ dependency shown in Oefner *et al*., [7], Glossop et al.,[42]). In the crystal structure of the NEPv presented here, the two mutations have reduced the substrate binding sites S1’ and S2’ as well as decreased the binding site plasticity. While we cannot fully deconvolute the contributions of the individual mutants to these changes, it is likely that introduction of Lys at position 714 predominantly contributes to these local changes. This is supported by our observations that the largest change in specificity for a single mutant after the first round of screening was observed with G714K and the second largest effect for D709K.

One of the Aβ1–40 cleavage fragments produced by wild-type NEP is Asp^1^-Phe^19^. This fragment is not observed as a NEPv cleavage product, which may partly be explained by the altered and reduced S1’ pocket. The affinity of the P1’ phenylalanine for the S1’ pocket is most likely decreased and cleavage at Phe^19^-Phe^20^ is less likely to occur. Furthermore, the electrostatic interactions on the prime side of the cleavage site are changed due to the introduction of the charged Lys^714^ in the S2’ pocket, and conformational changes of e.g. Arg^102^, Arg^110^ and Phe^106^. It could be envisaged that these changes favour cleavage at Phe^20^-Ala^21^ with a Glu at the P2’ position. This is supported by the recent modelling of Aβ interactions with wild-type NEP [43]. The model suggests a strong interaction between Glu^22^ of Aβ with the side chains of Arg^102^ and Arg^110^ of NEP. Our data indicating large conformational changes in the orientation of these side chains would be consistent with a change in the preferences for amino acids in the P2’ position.

In the same way that the reduced size and plasticity of the substrate binding sites may affect the Aβ1–40 cleavage specificity, the mutations may affect specificity towards other natural substrates of NEP. Most notable is the reduced catalytic efficiency on bradykinin compared to wild-type NEP, which in part might originate from steric hindrance of the bradykinin P1’ phenylalanine and the NEPv S’ site.

However, as an additive effect is seen on specificity and catalytic efficiency with both mutations present, and the NEP single mutant G714K showed some Aβ1–40 cleavage at Phe^20^-Aal^21^, but preferentially cleaved at Leu^17^-Val^18^, other additional factors must play a role. The mutational effects on NEP conformation extend from the S1’ pocket out to the surface of the protein. To our knowledge, there is no public structural data available with information on substrate binding beyond the S2’ site. However, it is likely that the change in structure due to the mutations will further affect substrate binding specificity outside the S1’–S2’ region.

The size of the opening to, as well as the size of, the catalytic chamber has been noted [44] to be incompatible with diffusion and fitting of folded Aβ into the catalytic cavity. Two hypothetical conformational changes have been proposed to accommodate entry of large substrates into the active site: one where a localized loop shift allows substrate access, the other a hinge-mediated opening of domain 1 and 2. The related neutral endopeptidase thermolysin has been observed as both substrate analogue-bound closed [45] and unoccupied open [46] structures, and transition between these conformations, via hinge bending, is thought to occur during catalysis [47]. Recently, for insulin degrading enzyme, another member of the cryptidase family, both domain opening as well as localized loop-shifts have been observed, that allow substrate access to the active site [48]. The authors speculate that the substrate interaction with the open states contribute to substrate specificity. We have observed an alternative crystal form of NEPv, which showed a domain opening where increased access to the catalytic chamber is observed (manuscript in preparation). It is enticing to assume that the NEP variant has an altered substrate access dynamics; particularly the G399V mutation located at the domain interface could be involved in domain opening dynamics. This, in addition to the local changes in substrate binding sites, could contribute to the observed altered substrate specificity.

These hypotheses are further supported by the observation that NEPv had considerably reduced sensitivity compared to wild-type NEP to inhibition by thiorphan and phosphoramidon. While the crystal structure of phosphoamidon bound to NEPv shows only modest changes in the binding mode of the inhibitor, changed domain opening dynamics and the propensity of ligands to induce domain closure may govern the reduced inhibition observed by these small molecule inhibitors. Whereas these inhibitors effectively block the activity of wild-type NEP with low nM potencies, µM concentrations were required to have any effect on NEPv activity. The observations on the effects of the inhibitors provide further evidence that the mutations introduced into NEPv have substantially altered the active site binding selectivity of the enzyme.

Our engineering of NEP appears to have fundamentally changed the cleavage site preference of the enzyme, and it is possible that this change has increased cleavage activity on other peptides not tested here. The engineered variant of NEP described here has significantly increased activity on and specificity towards Aβ, and therefore has potential to be a more efficacious therapeutic for Alzheimer’s disease with less activity on other peptides than wild-type NEP. Further development of NEPv as a therapeutic may require analysis of the cleavage of a greater range of peptide substrates, although this was beyond the scope of the present study.

Recent studies have shown that reduction of peripheral Aβ through intravenous administration of NEP has no effect on brain Aβ levels, suggesting that a peripheral sink of the peptide that can be targeted therapeutically to reduce brain amyloid burden does not exist [49], [50]. Therefore, more targeted approaches, using either direct delivery into the brain or via blood brain barrier transport vectors, may be required for amyloid-reducing drugs to be effective.

## Supporting Information

Figure S1 **Degradation of Aβ1–40 and Aβ1–42 by NEP wild-type and NEPv.** Measurement of the degredation of Aβ1–40 (A) and Aβ1–42 (B) was performed by incubating peptide and either wild-type NEP (▪) or NEPv (Δ) for 1 hour at room temperature. The reaction was stopped by the addition of 1.10-phenanthroline to a final concentration of 10 µM. 50 µl (Aβ40 analysis) or 100 µl (Aβ42 analysis) of the reaction mix was transferred to an ELISA plate. Aβ40 and Aβ42 concentrations after degradation were determined using Invitrogen human Aβ40 ELISA kit (Invitrogen, California, US), and Innotest β-amyloid RUO (1–42) ELISA (Innogenetics, Gent, Belgium). EC50s from the curves were calculated as: NEP Aβ1–40, 2.433×10^−7^, Aβ1–42, 1.442×10^−7^, NEPv Aβ1–40, 2.126×10^−8^, and Aβ1–42, 1.945×10^−8^, indicating equivalence of activity on either peptide.
(TIF)

Figure S2 **Inhibition of wild-type NEP and mutants by phosphoramidon and thiorphan.** Aβ1**–**40 cleavage activity was determined for wild-type NEP (○), NEP G399V (▪), NEP G714K (*) and NEPv (▾) in the presence of a range of concentrations of phosphoramidon (A) or thiorphan (B) in assays containing 20****nM enzyme and 10** µ**M substrate. Activity data were normalised to that of the uninhibited control and plotted against log_10_[inhibitor], and a log[inhibitor] vs. response equation was used to fit them. Error bars represent the spread of two duplicate data points and the plots shown are representative of three replicate experiments. The compounds inhibited wild-type NEP>NEP G714K>NEP G399V>NEPv.
(TIF)

Figure S3 **2fo-fc electron density maps for (A) Val399 and (B) Lys714.**
(TIF)

Table S1 **Sequences of substrates used peptide cleavage assays.** ^a^Peptide substrates used for screening NEP variants. Peptides were labelled at the N-terminus with DY505 or DY647 and at the C-terminus with biotin. ^b^Peptide substrates used for detailed kinetic characterisation of NEP variants. Peptides were labelled at the N-terminus with 5(6)FAM and at the C-terminus with biotin. ^c^Unlabelled endothelin-1 used for determination of kinetic parameters in HPLC assay. ^d^Modified endothelin-1 contained an additional N-terminal Gly residue had 1–15 or 3–11 disulphide bond removed to improve peptide solubility when modified with fluorescent dye and biotin. ANP = atrial natriuretic peptide; BNP = brain natriuretic peptide; GLP-1 = glucagon-like peptide-1; GRP = gastri-releasing peptide.
(DOCX)

Table S2 **IC_50_ values for inhibition of wild-type NEP and mutants by phosphoramidon and thiorphan.** ^a^IC_50_ values were determined by measuring Aβ1–40 cleavage activity with a substrate concentration of 10 µM, a NEP concentration of 20 nM and inhibitor concentrations between 2 nM and 100 µM. Values are means from three replicate experiments and are quoted ± S.E.M. ^b^Due to the relatively low activity of wild-type NEP on Aβ1–40, the lowest enzyme concentration that could be used in the assay was 20 nM, and therefore it was not possible to determine IC_50_<10 nM.
(DOCX)
