# Supplementary figures and images for: Engineering Neprilysin Activity and Specificity to Create a Novel Therapeutic for Alzheimer’s Disease
Source: PLoS One. 2014 Aug 4;9(8):e104001. doi: 10.1371/journal.pone.0104001 (PMC4121237; doi:10.1371/journal.pone.0104001)

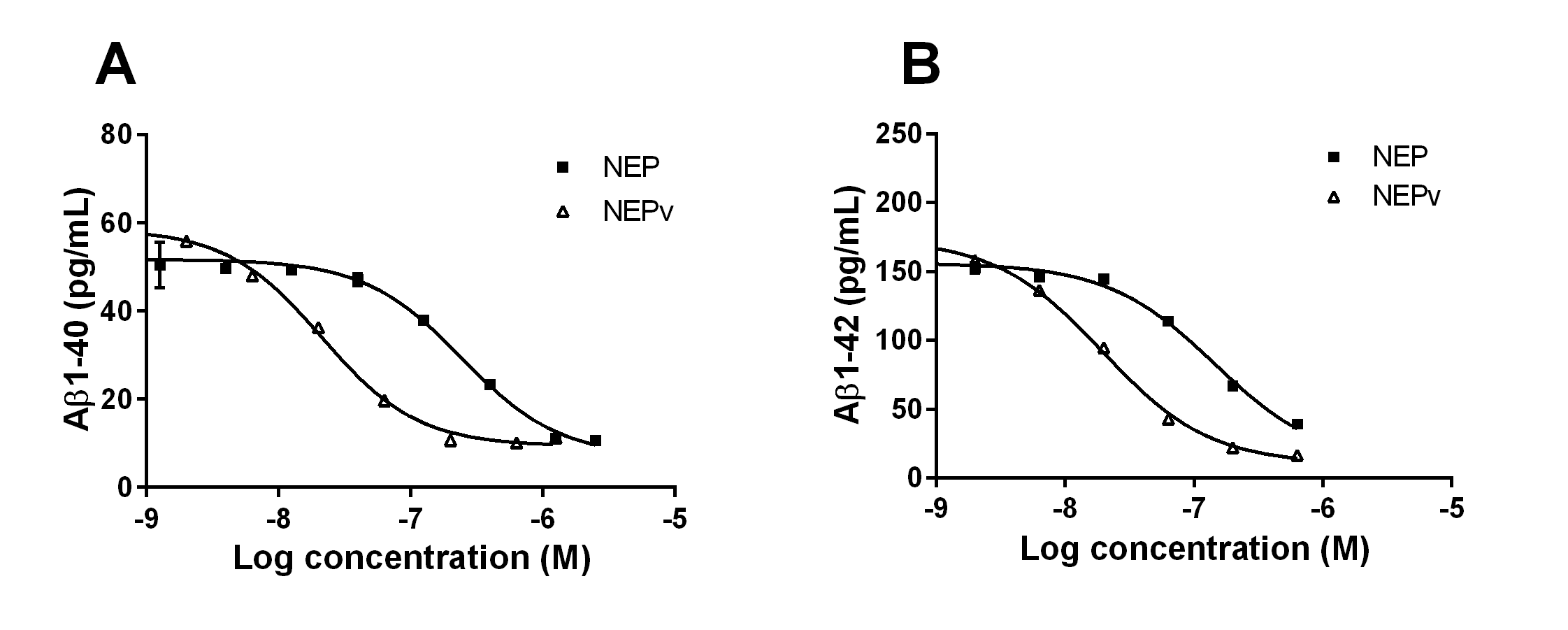

Supplement: Figure S1 — Degradation of Aβ1–40 and Aβ1–42 by NEP wild-type and NEPv. Measurement of the degredation of Aβ1–40 (A) and Aβ1–42 (B) was performed by incubating peptide and either wild-type NEP (▪) or NEPv (Δ) for 1 hour at room temperature. The reaction was stopped by the addition of 1.10-phenanthroline to a final concentration of 10 µM. 50 µl (Aβ40 analysis) or 100 µl (Aβ42 analysis) of the reaction mix was transferred to an ELISA plate. Aβ40 and Aβ42 concentrations after degradation were determined using Invitrogen human Aβ40 ELISA kit (Invitrogen, California, US), and Innotest β-amyloid RUO (1–42) ELISA (Innogenetics, Gent, Belgium). EC50s from the curves were calculated as: NEP Aβ1–40, 2.433×10^−7^, Aβ1–42, 1.442×10^−7^, NEPv Aβ1–40, 2.126×10^−8^, and Aβ1–42, 1.945×10^−8^, indicating equivalence of activity on either peptide. (TIF) [file pone.0104001.s001.tif]

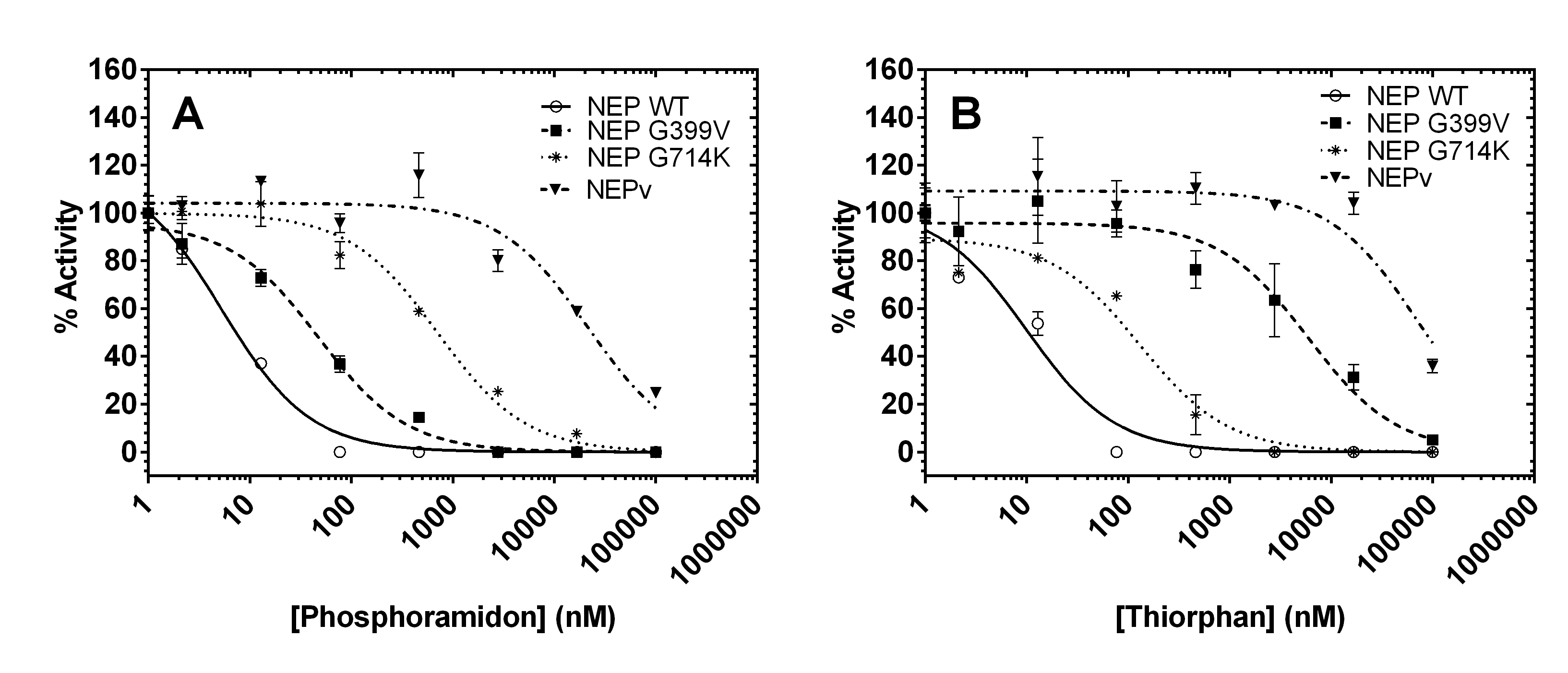

Supplement: Figure S2 — Inhibition of wild-type NEP and mutants by phosphoramidon and thiorphan. Aβ1–40 cleavage activity was determined for wild-type NEP (○), NEP G399V (▪), NEP G714K (*) and NEPv (▾) in the presence of a range of concentrations of phosphoramidon (A) or thiorphan (B) in assays containing 20nM enzyme and 10 µM substrate. Activity data were normalised to that of the uninhibited control and plotted against log10[inhibitor], and a log[inhibitor] vs. response equation was used to fit them. Error bars represent the spread of two duplicate data points and the plots shown are representative of three replicate experiments. The compounds inhibited wild-type NEP>NEP G714K>NEP G399V>NEPv. (TIF) [file pone.0104001.s002.tif]

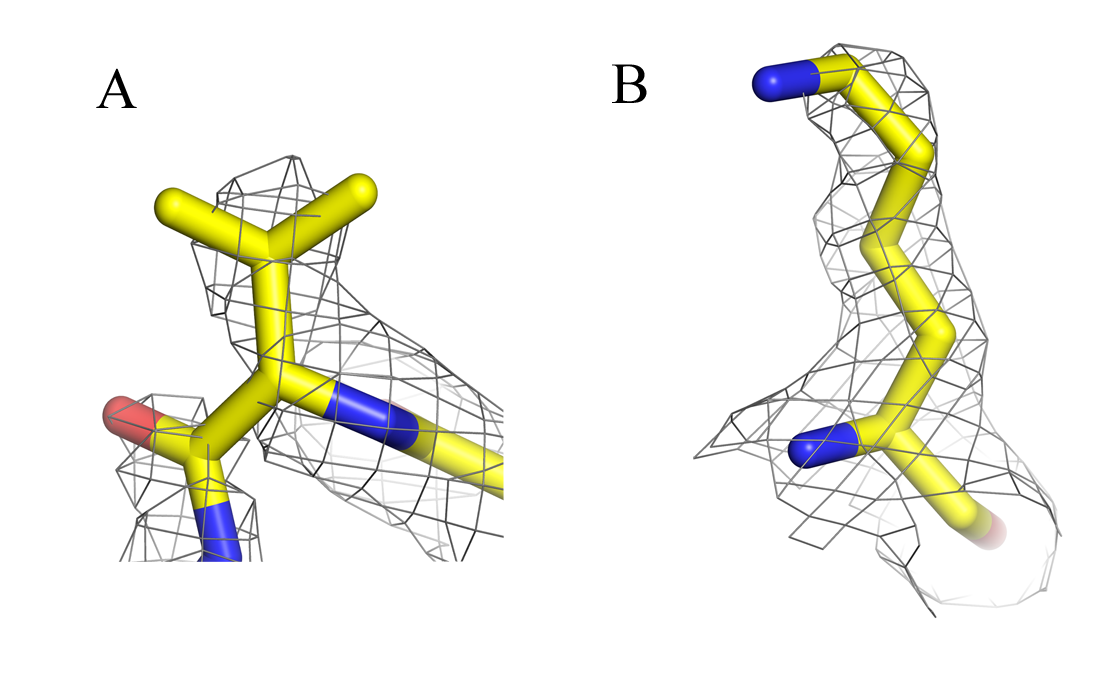

Supplement: Figure S3 — 2fo-fc electron density maps for (A) Val399 and (B) Lys714. (TIF) [file pone.0104001.s003.tif]
