# Supplementary material for: Engineering Neprilysin Activity and Specificity to Create a Novel Therapeutic for Alzheimer’s Disease
Source: PLoS One. 2014 Aug 4;9(8):e104001. doi: 10.1371/journal.pone.0104001 (PMC4121237; doi:10.1371/journal.pone.0104001)
Supplement: Table S1 — Sequences of substrates used peptide cleavage assays. aPeptide substrates used for screening NEP variants. Peptides were labelled at the N-terminus with DY505 or DY647 and at the C-terminus with biotin. bPeptide substrates used for detailed kinetic characterisation of NEP variants. Peptides were labelled at the N-terminus with 5(6)FAM and at the C-terminus with biotin. cUnlabelled endothelin-1 used for determination of kinetic parameters in HPLC assay. dModified endothelin-1 contained an additional N-terminal Gly residue had 1–15 or 3–11 disulphide bond removed to improve peptide solubility when modified with fluorescent dye and biotin. ANP = atrial natriuretic peptide; BNP = brain natriuretic peptide; GLP-1 = glucagon-like peptide-1; GRP = gastri-releasing peptide. (DOCX) [file pone.0104001.s004.docx]

| **Peptide** | **Sequence** |
| --- | --- |
| Aβ 1-40^a,b^ | DAEFRHDSGYEVHHQKLVFFAEDVGSNKGAIIGLMVGGVV |
| Aβ 1-42^a^ | DAEFRHDSGYEVHHQKLVFFAEDVGSNKGAIIGLMVGGVVIA |
| Neurotensin^a,b^ | QLYENKPRRPYIL |
| ANP^a,b^ | SLRRSSCFGGRMDRIGAQSGLGCNSFRY |
| BNP^a^ | SPKMVQGSGCFGRKMDRISSSSGLGCKVLRRH |
| Endothelin-1^c^ | CSCSSLMDKECVYFCHLDIIW |
| Modified Endothelin-1^a,d^ | a) GCSSSSLMDKESVYFCHLDIIWK  b) GSSCSSLMDKECVYFSHLDIIWK |
| Neuropeptide Y^a^ | CYPSKPDNPGEDAPAEDMARYYSALRHYINLITRQRY |
| Insulin B-chain^a^ | FVNQHLCGSHLVEALYLVCGERGFFYTPKT |
| Angiotensin^a,b^ | DRVYIHPFHL |
| Bradykinin^a,b^ | RPPGFSPFR |
| GLP-1^b^ | HAEGTFTSDVSSYLEGQAAKEFIAWLVKGRG |
| Glucagon^b^ | HSQGTFTSDYSKYLDSRRAQDFVQWLMNT |
| Somatostatin 28^b^ | SANSNPAMAPRERKAGCKNFFWKTFTSC |
| Somatostatin 14^b^ | AGCKNFFWKTFTSC |
| Neurokinin A^b^ | HKTDSFVGLM |
| Neurokinin B^b^ | DMHDFFVGLM |
| Nociceptin^b^ | FGGFTGARKSARKLANQ |
| α-endorphin^b^ | YGGFMTSEKSQTPLVT |
| γ-endorphin^b^ | YGGFMTSEKSQTPLVTL |
| Substance P^b^ | RPKPQQFFGLM |
| Arg-vasopressin^b^ | CYFQNCPRG |
| GRP^b^ | VPLPAGGGTVLTKMYPRGNHWAVGHLM |
