# Supplementary material for: Engineering Neprilysin Activity and Specificity to Create a Novel Therapeutic for Alzheimer’s Disease
Source: PLoS One. 2014 Aug 4;9(8):e104001. doi: 10.1371/journal.pone.0104001 (PMC4121237; doi:10.1371/journal.pone.0104001)
Supplement: Table S2 — IC50 values for inhibition of wild-type NEP and mutants by phosphoramidon and thiorphan. aIC50 values were determined by measuring Aβ1–40 cleavage activity with a substrate concentration of 10 µM, a NEP concentration of 20 nM and inhibitor concentrations between 2 nM and 100 µM. Values are means from three replicate experiments and are quoted ± S.E.M. bDue to the relatively low activity of wild-type NEP on Aβ1–40, the lowest enzyme concentration that could be used in the assay was 20 nM, and therefore it was not possible to determine IC50<10 nM. (DOCX) [file pone.0104001.s005.docx]

| **Variant** | **IC_50_ (nM)^a^** | |
| --- | --- | --- |
|  | Phosphoramidon | Thiorphan |
| Wild-type NEP^b^ | <10 | <10 |
| NEP G399V | 610 ± 90 | 4400 ± 1000 |
| NEP G714K | 100 ± 40 | 75 ± 20 |
| NEPv | 21000 ± 1000 | 77000 ± 3000 |
